# Correlates of social behavior change communication on care-seeking behaviors for children with fever: an analysis of malaria household survey data from Liberia

**DOI:** 10.1186/s12936-018-2249-x

**Published:** 2018-03-07

**Authors:** Grace Awantang, Stella Babalola, Hannah Koenker, Kathleen Fox, Michael Toso, Nan Lewicky, Daniel Somah, Victor Koko

**Affiliations:** 1 Health Communication and Capacity Collaborative Project, Johns Hopkins Center for Communication Programs, 111 Market Place, Suite 310, Baltimore, MD 21202 USA; 2 National Malaria Control Programme, Ministry of Health, Capitol By-Pass, PO Box 10-9009, 1000 Monrovia 10, Liberia

**Keywords:** Care seeking, Treatment, Social and behavior change communication, Campaign, Malaria

## Abstract

**Background:**

In 2010, malaria was responsible for an estimated 41% of deaths among children under the age of five years in Liberia. The same year, the Rebuilding Basic Health Services Project launched “Healthy Baby, Happy Mother,” a social and behavior change communication campaign. The campaign encouraged caregivers to take children under the age of five years to a health facility as soon as children developed fever. This study investigated correlates of two case management outcomes: care-seeking for children under five with fever during the past two weeks and administration of an artemisinin-based combination therapy (ACT) the same or next day as fever onset.

**Methods:**

Data from a 2014 cross-sectional household survey from four counties was used to investigate correlates of two case management outcomes. Using multilevel analysis, the association between these outcomes and a caregiver’s recall of the campaign, her sociodemographic characteristics, and unmeasured characteristics of the community she lived in was investigated.

**Results:**

Caregivers living in Grand Kru County were less likely (OR = 0.21, 95% CI 0.073, 0.632) to take a child to a health facility than those in Bong County. Caregiver recall of the campaign was positively associated with the odds that a child received an ACT promptly (OR 3.62, 95% CI 1.398–9.372), but not with the odds of a caregiver taking a child in their care to a health facility. While unmeasured community-level factors accounted for 19.0% of the variation in the odds that a caregiver’s child was brought to a health facility, they did not play a role in the odds of prompt ACT treatment.

**Conclusions:**

Recalling the “Healthy Mother, Happy Baby” campaign was positively associated with the odds that children received ACT promptly, even in the absence of other malaria prevention and treatment messaging. While caregiver exposure was not associated with care-seeking during the two weeks before interview, prompt care-seeking likely preceded prompt receipt of ACT since most ACT came from health facilities. Unmeasured community-level factors, such as distance from the health facility, may play a role in determining the odds that a caregiver takes a child to a health facility.

## Background

In Liberia, an estimated 41% of deaths among children under age five were attributed to malaria in 2010 [1]. In its 2010–2015 national malaria strategic plan, the Liberian Ministry of Health and Social Welfare [now the Ministry of Health (MOH)] aimed to ensure early diagnosis and prompt and effective treatment of uncomplicated malaria among 80% of patients by the end of 2010 and sustain this through 2015. The plan identified children under five and pregnant women as particularly vulnerable populations to be addressed [1]. However, according to the 2011 Malaria Indicator Survey, three-fifths (59.7%) of Liberian children under the age of five years received care at a health facility and about two-fifths (39.7%) [2] of children who developed fever were given artesunate–amodiaquine (ASAQ) or another artemisinin-based combination, as recommended for uncomplicated malaria [1]. The World Health Organization recommends treatment for children under five within 24 h of symptom onset. However, the prevalence of prompt artemisinin-based combination therapy (ACT) among children in Liberia was stagnant for several years [2, 3] before this study took place in 2014. Liberia’s case management strategy incorporates both facility- and community-level interventions [1], as ACT has historically only been available at health facilities [1]. Both malaria diagnosis and ACT are free at public health facilities [4].

To help reduce the health toll caused by malaria, the MOH and the JSI Research and Training Institute, Jhpiego, Management Sciences for Health, and the Johns Hopkins Center for Communication Programs collaborated to implement the Rebuilding Basic Health Services (RBHS) project from 2010 to 2014, with the financial support of USAID. In October of 2010, the RBHS project launched the “Healthy Baby, Happy Mother” social and behavior change communication (SBCC) campaign to complement supply-side components of the project [5]. Using radio and various print materials that RBHS distributed through government health facilities, general community health volunteers (gCHVs), and schools, the “Healthy Baby, Happy Mother” campaign disseminated four key messages about how a caregiver should respond if her child developed fever. One of these key messages encouraged caregivers to immediately seek care at a health facility should a child develop fever.

Understanding what factors prevent or facilitate a child’s likelihood of being taken to a health facility and receiving ACT treatment promptly is crucial to helping the MOH advance strategies to improve case management outcomes across the country. This paper investigates the correlates of two different malaria case management outcomes: (1) a female caregiver taking a child to a health facility and (2) the prompt treatment of febrile children with ACT. The authors were specifically interested in caregiver exposure to “Healthy Baby, Happy Mother.” One study has documented the effectiveness of SBCC as a means of changing care-seeking behavior for children under five with fever in India [6]. Aside from Kenny et al. [7], no other studies examine correlates of a caregiver seeking care for a child with fever from a health facility in Liberia. Similarly, apart from Shah et al. [8], this is the only study that has examined the correlates of prompt treatment with ACT among children in Liberia.

### Social behavior change communication campaign

In partnership with the National Malaria Control Programme and the National Health Promotion Division of the MOH, the RBHS project developed early case management materials and audio messages as part of the “Healthy Baby, Happy Mother” campaign [5]. Campaign materials included five radio spots promoting four key case management messages that encouraged early detection of child fever, home management of child fever, prompt care-seeking at a health facility, and full treatment compliance according to the directions of a health provider [9]. Case management radio messages were aired on two national radio stations and on eight community-based radio stations [9] in the five project counties (Marietta Yekee, personal communication January 23, 2017). Between October 2010 and October 2014, the radio spots were aired 7776 times—a total of 129.6 playing hours [9]. In addition, posters and brochures were distributed in two of the study counties: Bong and Grand Cape Mount. Approximately 30,000 posters and 50,000 brochures were distributed to health facilities, gCHVs, and school children [5] in five total counties, including Bong and Grand Cape Mount. The brochure specified that ACT was a ‘new’ medicine in Liberia that treated malaria. The poster focused on home management and instructed caregivers to give a febrile child paracetamol (acetaminophen) and a cold sponge bath and promptly seek care at a health facility.

## Methods

### Sampling

The data analysed in this manuscript came from a 2014 cross-sectional household survey implemented to inform malaria prevention and treatment programmes in Liberia. For this survey, all 15 Liberian counties were separated into a low- or high-malaria transmission group based on whether the prevalence of parasitaemia among children 6–59 months of age was below or above the median county prevalence (52%) [10]. Two counties from each group were then selected at random. The selected counties from the lower prevalence group were Bong (41%) and River Cess (50%), and the counties from the higher prevalence group were Cape Mount (59%) and Grand Kru (70%). Within each county, 15 clusters or villages were selected based on their population size, as reported in the 2008 National Population and Housing Census data. Female caregivers from 600 households in each transmission group were interviewed in order to detect differences between malaria prevention and treatment behaviors across levels of malaria transmission of 10% points or greater. In each cluster, 20 households that were home to children under five years old were selected to participate in the survey. One female caregiver from each household was interviewed.

### Data collection

In 2014, Subah-Belleh Associates, a local research firm, collected data from 1200 households (300 from each county) and interviewed 1200 female caregivers between March 31 and April 22, 2014. Only households that were home to a child under five who was under the care of a female caregiver were eligible for the interview. In each eligible house, after obtaining informed consent, interviewers approached the head of the household and asked him or her list all of the people who slept in the household the night before the interview. Data collectors also used the household questionnaire to record sociodemographic characteristics of the household and information about the household’s physical dwelling. From the household listing, data collectors randomly selected a child between the ages of 0 and 4 years old and interviewed the child’s primary female caregiver using an individual questionnaire. Depending on the composition of a household, the head of the household was sometimes also the caregiver who responded to the individual questionnaire; in other words, a single individual could have completed both questionnaires.

The individual questionnaire was used to gather information about the female caregiver and if her child had recently had fever. If her child had been ill with fever, she was asked if and where she sought care, whether or not the child received a blood or finger stick for malaria diagnosis, and what medications the child was given for fever, if any. Interviewers asked caregivers to indicate if a child in their care had been given sulfadoxine–pyrimethamine, chloroquine, quinine, ASAQ, aspirin, paracetamol, or any other drugs for their fever. If a caregiver’s child had suffered from fever recently and had received ASAQ, interviewers asked her if the child received ASAQ the same or next day as the fever’s onset, to determine whether an ACT had been received promptly. Interviewers did not ask caregivers if they had sought care the same or next day as fever onset. Interviewers also asked caregivers about their exposure to any malaria prevention or treatment message as well as to the “Healthy Baby, Happy Mother” campaign, and what campaign messages they recalled. Data from the hard copy questionnaires were entered into a Census and Survey Processing System data shell.

### Analysis

Analysis was limited to 607 female caregiver and child pairs in which the child had experienced fever within the two weeks prior to data collection. The authors used Stata (version 14.0) to analyse all data. First, all caregivers were nested within their sampling cluster or community and ran bivariate analysis to examine how each potential correlate was associated with the two dependent outcomes. Using the variables that were significant from this step, multivariate multilevel models were run to detect association with the two dependent variables. For each dependent variable, an empty model was run with no covariates to assess the variance in the outcome at the cluster level. Based on significance of independent variables in bivariate analysis, a model was constructed for each dependent variable by adding the same set of independent variables to a full model.

### Dependent variables

While the campaign encouraged caregivers to seek care promptly at a health facility, the questionnaire design prohibited an investigation of the relationship between a caregiver’s exposure to the campaign and prompt care-seeking at a health facility for her child’s fever. The reason for this was that the individual questionnaire asked caregivers if they had taken their child with fever in the last two weeks to a health facility, but not if they had taken the child immediately after the child had become ill. In contrast, the questionnaire did ask the caregivers if the child had received an ACT and then asked them if this had occurred the same or next day as fever onset. In order to examine prompt care-seeking behavior among female caregivers, this analysis examined correlates of a caregiver taking her child with fever during the last two weeks to a health facility and correlates of that child receiving ACT the same or next day as fever onset. Two binary dependent variables of interest were created to determine if a woman sought care at a health facility at any time during a child’s fever and whether or not a caregiver’s child receiving ASAQ or an ACT the same or next day as fever onset. Caregivers who sought care at a hospital, community health center, health post, mobile clinic, or pharmacy were considered as having sought treatment at a health facility.

### Independent variables

Independent variables included a woman’s educational level, religion, age, marital status, household size, household’s wealth quintile, county of residence, exposure to radio, recall of campaign messages, and recall of any malaria prevention or treatment message. Wealth quintiles were constructed based on household characteristics and ownership of assets. Each head of household described their household in terms of various physical characteristics, such as the type of roof, floor, and water infrastructure. Principal component analysis was used to categorize each household as belonging to one of five wealth quintiles, with each quintile reflecting the relative wealth of each household compared to all households sampled. To account for the possible influence of other anti-malarial communication interventions, interviewers first asked caregivers if they had heard or seen any malaria prevention or treatment messages in the past year. To measure exposure to the campaign, interviewers also asked caregivers if they had heard or seen the phrase “Healthy Baby, Happy Mother” in the last 6 months. Interviewers then asked caregivers how they had been exposed to this campaign. Possible responses included radio, posters, television, community events, a friend/family/neighbor, and a health-care worker. The last response potentially included not only facility-based health providers, but also gCHVs. Next, interviewers asked exposed caregivers to recall what health messages they associated with the phrase. A categorical variable was created to assign all female caregivers to one of four groups according to their ability to recall anti-malarial and/or campaign messages. The first group was comprised of female caregivers who were not able to recall any campaign messages or any messages relating to malaria prevention and treatment. The second group included caregivers who could not recall any campaign messages, but recalled hearing or seeing a message relating to malaria prevention and treatment. The third group included caregivers who could recall one or more campaign messages, but said they had not heard or seen any anti-malarial prevention and treatment messages. The fourth group included caregivers who recalled one or more campaign messages and reported hearing or seeing an anti-malarial prevention or treatment messages.

## Results

### Respondent characteristics

Table 1 presents characteristics of interviewed female caregivers whose children had been sick with fever during the past two weeks and caregivers whose children had not. The proportion of caregivers between the ages of 18 and 24 years old was smaller among caregivers with sick children (29.0%) than those without (38.4%). Over half of caregivers with sick children (55.5%) and without (54.5%) had either no education or had not completed primary school. Roughly three-quarters (76.1%) of the caregivers with sick children were Christian and almost one-fifth (19.0%) were Muslim. Among caregivers without sick children the proportions reporting different religions varied. Most of the female caregivers (86.8%) with sick children and without sick children (83.3%) were married. About three-fifths (60.5%) of the caregivers with sick children listened to the radio at least once a week, while a smaller proportion of caregivers (45.9%) without sick children listened to the radio at least once a week. The distribution of caregivers with and without children varied across the four study counties. The size of caregivers households varied between those with caregivers with and without sick children in the past two weeks.

**Table 1:** Characteristics of female caregivers with and without a febrile child under five in the last two weeks

	Child had fever in past two weeks	Child did not have fever
	%/(n)^a^	%/(n)^b^
Mean age in years		
18–24	29.0 (76)	38.4 (214)
25–29	24.2 (147)	22.4 (125)
30–34	17.6 (107)	16.0 (89)
35–39	14.3 (87)	11.7 (65)
40–85	14.8 (90)	11.7 (65)
Education		
None	55.5 (337)	54.5 (304)
Primary school	34.9 (212)	32.7 (188)
Secondary school	9.6 (58)	11.8 (66)
Caregiver’s religion		
Other	4.9 (30)	1.4 (8)
Muslim	19.0 (115)	24.9 (139)
Christian	76.1 (462)	73.7 (411)
Caregiver’s marital status		
Non-married	13.2 (80)	16.7 (93)
Married	86.8 (527)	83.3 (465)
Listens to radio weekly		
Yes	39.5 (240)	54.1 (302)
No	60.5 (367)	45.9 (256)
County of residence		
Bong	16.6 (95)	34.2 (191)
Cape Mount	20.8 (119)	29.6 (165)
Grand Kru	34.0 (195)	15.4 (165)
River Cess	28.6 (164)	20.8 (116)
Household wealth		
Lowest	23.4 (142)	16.7 (93)
Second	20.4 (124)	19.4 (108)
Middle	19.6 (119)	21.0 (117)
Fourth	17.8 (108)	21.7 (121)
Highest	18.8 (114)	21.3 (119)
Household size	5.7 (2–19)	
Two to four	32.6 (198)	46.2 (258)
Five	23.2 (141)	22.6 (126)
Six	15.0 (91)	11.7 (65)
Seven to 19	29.2 (177)	19.5 (109)

^a^N = 607, ^b^N = 558

Table 2 describes caregiver exposure to anti-malarial messages as well as the “Healthy Baby, Happy Mother” campaign. Among all the female caregivers, about a third (35.4%) were either not exposed to or could not recall any health messages associated with the “Healthy Baby, Happy Mother” campaign (results not shown in Table 2). About one-tenth (11.2%) of caregivers did not report hearing or seeing a malaria prevention or treatment message in the past year or hearing or seeing the “Healthy Baby, Happy Mother,” campaign in the past 6 months. Slightly more (12.2%) had heard or seen a malaria prevention or treatment message in the past year, but had not heard or seen the phrase “Healthy Baby, Happy Mother.” A small proportion (5.1%) of caregivers had heard or seen the campaign phrase, but reported not hearing or seeing malaria prevention or treatment message. Most commonly (71.5%), caregivers reported hearing or seeing the phrase “Healthy Baby, Happy Mother” in the 6 months before their interview and having seen a malaria prevention or treatment message. The most common channels through which women were exposed to the campaign were the radio (59.4%) and health-care workers (57.8%)—a category that included both facility-based health workers and gCHVs. Caregiver exposure to the campaign through other communication channels, such as posters (26.2%); discussions with friends, neighbors, or family members (18.9%); and community events (14.2%), was less common. In terms of how exposure to the campaign channels varied across counties, the proportion of exposed caregivers who heard the phrase, “Healthy Baby, Happy Mother” on the radio varied across River Cess (69.6%), Bong (62.2%), Grand Cape Mount (50.0%), and Grand Kru (50.0%) counties. The proportion of exposed caregivers who saw the campaign phrase on a poster was greater in Bong (36.7%) and Grand Cape Mount (55.9%) counties than in Grand Kru (7.7%) and River Cess (13.0%), which were counties where the poster was not widely distributed (results not shown in Table 2). Exposed caregivers recalled an average of 1.4 of the four campaign case management messages (results not shown in Table 2). Among exposed women, the most commonly recalled message was taking a full dose of treatment for malaria (60.0%). A third of exposed caregivers recalled the message that instructed mothers to seek care promptly for a child’s fever (33.1%), give the child a sponge bath before seeking care (28.0%), and detect fever early (15.3%).

**Table 2:** Message exposure characteristics of caregivers with a febrile child under five

	% (n)
Did not hear or see malaria prevention or treatment message nor “Healthy Baby, Happy Mother”	11.2 (68)
Heard or saw malaria prevention or treatment message in past year,^a^ but not “Healthy Baby, Happy Mother”	12.2 (74)
Heard or saw the phrase “Healthy Baby, Happy Mother” in past 6 months^b^ but not a malaria prevention or treatment message	5.1 (31)
Heard or saw both “Healthy Baby, Happy Mother” and a malaria prevention or treatment message	71.5 (434)
Sources of exposure among exposed caregivers^b^	
Radio	59.4 (276)
Television	0.22 (1)
Poster	26.2 (122)
Community event	14.2 (66)
Healthcare worker	57.8 (269)
Neighbor, friend or family member	18.9 (88)
Campaign messages recall among exposed caregivers^b^	
Detect fever early	15.3 (72)
Seek prompt treatment for fever	33.1 (154)
Take the full dose of treatment for malaria	60.0 (279)
Give child a sponge bath before seeking care	28.0 (130)

Baby, Happy Mother” in the past 6 months

^a^N = 607, all caregivers with a febrile child

^b^N = 465, caregivers with a febrile child who were exposed to “Healthy

Ideally, children taken to a health a facility would be tested for malaria and prescribed an ACT if the child was diagnosed with malaria. Results indicated the vast majority (98.5%) of female caregivers sought advice or treatment for children who had suffered from fever within the two weeks before the survey interview. The two most common places caregivers sought out treatment were at a government hospital (52.1%) or community health center (20.7%). Caregivers less frequently sought treatment for child fever from a travelling medicine seller (8.5%), pharmacy (7.7%), private hospital/clinic (7.5%), or traditional health practitioner (6.0%). Table 3 displays the prevalence of various care-seeking, diagnosis, and treatment behaviors among interviewed caregivers. Four-fifths (80.6%) of sick children were taken to a health facility. Nearly three-quarters (72.6%) of children received a blood test for malaria—likely using either an RDT or microscopic examination of a blood smear. Roughly three-fifths (60.4%) of children received a positive malaria diagnosis.

**Table 3:** Respondent care-seeking behaviors for febrile children whose caregivers sought treatment or advice for them

Proportion of children that…	% (n)^a^
Went to a health facility	80.6 (482)
Had a heel or finger blood sick	72.6 (434)
Test indicated malaria	60.4 (361)
Took medication for their fever	95.8 (573)
Took an ACT	66.7 (399)
Took an ACT the same or next day as fever onset	38.0 (227)

Although taking medication is listed below malaria diagnosis and health facility consultation, children may have received medication before arriving at a health facility or receiving diagnosis

^a^N = 598

Almost all children (95.8%) for whom treatment or advice was sought were given medicine for their illness. Two-thirds (66.7%) of children received an ACT, however, only 38.0% were given the medication on the same day or the day following the fever’s onset. The most common places caregivers obtained ACT were at a government hospital (55.4%) or at a community health center (18.0%), a category that included community-based centers and clinics. Roughly fourth-fifths (86.2%) of caregivers whose children received an ACT also reported obtaining the medication from a government hospital or community health center. Caregivers less frequently sought out treatment for child fever from a family or friend (7.8%), traveling medicine seller (6.5%), different public health-care source (6.3%), or private hospital/clinic (6.0%) (not shown). Results indicated that a greater number of children received medication for fever than were taken to a health facility or diagnosed for malaria. Over two-fifths (43.1%) of children who received an ACT did not take it promptly.

### Multilevel analysis

Table 4 displays the results of multilevel analysis for the two behavioral outcomes. The interclass correlation coefficient (ICC) of 0.42 in the empty or null model (Table 4, Model 1) indicated that over two-fifths (42%) of the variance in a caregiver taking a child with fever to a health facility was due to unmeasured correlates. The full model (Table 4, Model 2) with all potential correlates indicated that a caregiver’s residence in Grand Kru County (OR 0.21, 95% CI 0.073, 0.632). The ICC for this model indicates that 19% of the variation in odds of seeking care at a health facility was dependent on correlates operating at the cluster or community level, such as distance from a health facility.

**Table 4:** Correlates of two care-seeking outcomes among female caregivers for child with fever

	Caregiver sought care at health facility			Child took ACT promptly		
	(1)^a^	(2)^a^		(3)^a^	(4)^a^	
		OR	95% CI		OR	95% CI
Caregiver’s current age in years		1.02	0.992, 1.058		1.00	0.983, 1.025
Caregiver’s education level						
None (RC)		1.00			1.00	
Primary		1.37	0.778, 2.427		0.98	0.655, 1.474
Secondary		2.27	0.878, 5.849		1.04	0.547, 1.983
Caregiver’s religion						
Non-Christian (RC)		1.00			1.00	
Christian		1.28	0.586, 2.809		0.59	0.328, 1.066
Caregiver’s marital status						
Non-married (RC)		1.00			1.00	
Married		0.83	0.408,1.683		0.99	0.588, 1.658
Listens to radio weekly						
No (RC)		1.00			1.00	
Yes		1.59	0.952, 2.674		1.99***	1.359, 2.929
County of residence						
Bong (RC)		1.00			1.00	
Cape Mount		3.84	0.939, 15.722		0.69	0.337, 1.403
Grand Kru		0.21**	0.073, 0.632		0.53	0.350, 1.074
River Cess		1.06	0.353, 3.164		0.61	0.725, 2.113
Household wealth quintile						
Lowest (RC)		1.00			1.00	
Low		0.81	0.400, 1.631		0.61	0.350, 1.074
Middle		2.27	0.981, 5.272		1.24	0.725, 2.113
High		2.13	0.878, 5.187		1.07	0.606, 1.893
Highest		1.41	0.513, 3.868		0.96	0.525, 1.746
Household size		0.97	0.870, 1.078		1.03	0.948, 1.110
Message heard or seen						
Neither malaria prevention nor treatment message nor “Healthy Baby, Happy Mother” (RC)		1.00			1.00	
Malaria prevention or treatment message but not “Healthy Baby, Happy Mother”		1.88	0.767, 4.607		1.03	0.451, 2.353
Not Malaria prevention nor treatment message but “Healthy Baby, Happy Mother”		2.41	0.707, 8.194		3.62**	1.398, 9.372
Both malaria prevention or treatment message and “Healthy Baby, Happy Mother”		1.53	0.694, 3.354		2.11*	1.080, 4.131
Cluster level variance	2.31	0.77		0.17	0.00	
Intra-class correlation	0.42	0.19		0.05	0.00	
Log likelihood	− 272.4	− 247.1		− 399.8	− 374.3	
AIC	548.8	532.1		803.6	784.6	

RC denotes the reference category for each correlate

* p < 0.05; ** p < 0.01; *** p < 0.001

^a^N = 607

The second empty multilevel model (Table 4, Model 3), predicting whether a febrile child received ACT promptly, indicated that 17% of variance in this outcome was due to unmeasured differences at the community level. The full model (Table 4, Model 4) with all potential correlates indicated that listening to the radio at least once a week (OR 1.99, 95% CI 1.359, 2.929) was associated with increased odds of prompt ACT treatment. It also showed that recalling one or more campaign messages was significantly and positively correlated with increased odds of a caregiver’s child receiving ACT the same or next day as fever alone (OR 3.62, 95% CI 1.398, 9.372) or in combination (OR 2.11, 95% CI 1.090, 4.131) with hearing or seeing any malaria prevention or treatment message in the past year. Results indicated that children of caregivers who listened to the radio at least once a week were about twice as likely to receive an ACT promptly than those of caregivers who did not listen to the radio at least once a week. The ICC for this Model indicates that variation in the odds of prompt ACT at the community or cluster level was negligible.

## Discussion

No existing literature has described the correlation between caregiver exposure to SBCC campaign messages and caregivers seeking care at a health facility or their children receiving prompt ACT treatment in Liberia. Research has suggested that SBCC program exposure is associated with many malaria-related health behaviors, such as mosquito net use [11, 12] and anti-malarial treatment adherence in adults [13] elsewhere in sub-Saharan Africa. However, studies from this region have offered mixed evidence as to whether SBCC programs are positively associated with a caregiver’s care-seeking behaviors for child fever [14, 15]. These existing studies are of limited relevance because they examine the potential effects of SBCC interventions that differed greatly from the “Healthy Baby, Happy Mother” campaign [14] or because they do not isolate the role of the communication from structural supply-side interventions [15]. One of the two studies focused solely on an intervention that trained mothers to respond to child fever and did not include any community mobilization or mass media components [14]. The other study documented an increased likelihood of sick children receiving the recommended anti-malarial promptly [15] after a 3-year community-level SBCC intervention but did not observe an increase in health facility attendance [15]. Overall, no conclusive evidence has suggested that SBCC programs can influence care-seeking and treatment behaviors for child malaria in sub-Saharan Africa.

Results from this cross-sectional survey showed that a caregiver who recalled being exposed to the “Healthy Baby, Happy Mother” SBCC campaign message in the past 6 months was more likely to care for a febrile child that received ACT promptly—even in the absence of other malaria prevention and treatment messages. Although campaign exposure was not associated with seeking care at a health facility in general, it may have been associated with caregivers seeking care at health facilities promptly for two reasons. First, the sampled caregivers most commonly obtained ACT at health facilities, specifically government hospitals and community health centers. In this respect, the study sample was consistent with existing literature stating that ACT treatment was the recommended treatment at the time of data collection, but that ACT was mainly available at health facilities because of the complexity of their administration [1]. Second, the “Healthy Baby, Happy Mother” campaign scarcely mentioned ACT, with the exception of one mention in the campaign brochure. The four campaign radio spots, which were aired for years after the launch of the campaign, were the most common source of campaign exposure. These radio spots encouraged women to take their child to a health facility as soon as they detected a fever in their child, but did not mention ACT or suggest any other malaria drug. While proportion of caregivers who took their children to a health facility promptly could not be estimated, the “Healthy Baby, Happy Mother” campaign was successful in achieving high levels of exposure and the odds of caregivers taking a child with fever to a health facility the same or next day as the onset of fever were greater among exposed caregivers.

There was no significant association between the odds of a child’s health facility attendance and their female caregiver’s age, marital status, or level of education, unlike a study in Grand Gedeh County [7]. That study found that maternal education, age, marital status, and the age of the sick child were correlated with the odds of mothers seeking care for a child with fever at a health facility [7]. The relationship between media-use habits and care-seeking behavior has not been studied in Liberia. After controlling for exposure to the campaign, there was no significant link between listening to the radio every week and taking a child with fever to a health facility. Female caregivers in Malawi who listened to the radio daily were more likely to seek care for a febrile child at a hospital, rather than seeking traditional care or not seeking care at all [16].

Unmeasured community characteristics accounted for about a fifth of the variation in the odds of seeking care at a health facility. It is possible that a community’s distance from health facility [7] was among the sources of unmeasured variance influencing whether a female caregiver takes a child with fever to a health facility. The distance of a household from a health facility has been associated with decreased odds of seeking malaria treatment [17] and a delay in seeking care [18, 19] for febrile children in other sub-Saharan African countries. Similarly, the time needed to reach a facility, the availability of transport to reach the facility [16, 20] and perceptions of available care [21] have also been linked with whether a caregiver takes a child with fever to a health facility elsewhere in sub-Saharan Africa [16].

This study found that listening to the radio was positively associated prompt ACT administration. No available literature has investigated the association between a caregiver listening to the radio and the odds a caregiver’s child will promptly receive an ACT when febrile. Similar to an analysis of 2013 Liberian Demographic and Health Survey (DHS) data [8], there was no significant association between maternal age or maternal education and the likelihood of prompt ACT treatment among children with fever in the four study counties.

However, this study’s results differed from those of Shah and colleagues [8] in that household wealth was not correlated with either behavioral outcome. Using Liberia DHS 2013 data, Shah and colleagues found household wealth was the best predictor of a child receiving an ACT compared to any other drug [8]. They found that children in the lowest wealth quintile were more likely to receive prompt ACT treatment than those in the two highest wealth quintiles [8]. This is interesting in that diagnosis and treatment of fever in Liberia are generally free of cost [2, 4]. Evidence from other sub-Saharan African countries found that household wealth was not significantly related to caregivers delaying seeking care for children with fever [19].

The percentage of variation from unmeasured community-level factors contributing to the odds of receiving ACT promptly was much lower than the corresponding percentage for the odds of seeking care at a health-care facility.

### Limitations

This study has several limitations. All data is from a cross-sectional survey, which means associations did not signify causal attribution. The first Ebola cases were reported in Liberia during data collection and may have influenced the care-seeking behaviors for children with fever. However, relatively few suspected Ebola cases or confirmed deaths from Ebola were reported in April 2014 [22] when data collection took place. In addition, very few Ebola cases occurred in children under five (6%) [23]. The accuracy of the authors conclusions is subject to caregivers ability to recall campaign and the specific medicine that their child received. The study’s ability to describe variance in the two behavioral outcomes is limited by the omission of supply-side factors from the study questionnaires. As stated earlier, the study was not able to measure the campaign’s association with prompt care-seeking at a health facility—one of the campaign’s key target behaviors. Instead, this analysis examined similar case management behaviors: seeking care at a health facility and promptly receiving ACT.

## Conclusions

Social and behavior change communication campaigns that aim to promote desired care-seeking behaviors should be compelling enough for their audiences to remember as caregiver recall of messages is positively associated with positive care-seeking behaviors. Programmes designing SBCC campaigns should consider using radio to communicate with their audiences and should also consider supply-side barriers when crafting messages, since they help explain the occurrence of case management outcomes. Communication programs that generate demand for health-facility services will be most effective when coupled with interventions that address structural barriers to treatment of child malaria.

## Abbreviations

ACT: artemisinin-based combination therapy
ASAQ: artesunate–amodiaquine
DHS: Demographic and Health Survey
MOH: Ministry of Health
PMI: President’s Malaria Initiative
RBHS: Rebuilding Basic Health Services
RDT: rapid diagnostic test
SBCC: social and behavior change communication
USAID: United States Agency for International Development

## References

[1] Ministry of Health and Social Welfare National Malaria Control Programme (2010). National malaria strategic plan, 2010–2015.

[2] National Malaria Control Programme (NMCP), Ministry of Health and Social Welfare (MOHSW), Institute of Statistics and Geo-Information Services (LISGIS), ICF International (2011). Liberia malaria indicator survey 2011.

[3] Liberia Institute of Statistics and Geo-Information Services (LISGIS), Ministry of Health and Social Welfare National Malaria Control Programme, National AIDS Control Programme, ICF International (2013). Liberia demographic and health survey 2013.

[4] WHO (2010). World malaria report.

[5] JSI Research and Training Institute. Liberia: Rebuilding Basic Health Services (RBHS) Year 4 Annual Report. 1 October 2011–30 September 2012. Monrovia: JSI Research and Training Institute; 2012.

[6] Das A, Friedman J, Kandpal E, Ramana GNV, Gupta RKD, Pradhan MM (2015). Strengthening malaria service delivery through supportive supervision and communtiy mobilization in an endemic Indian setting: an evaluation of nested delivery models. Malar J.

[7] Kenny A, Basu G, Ballar M, Griffiths T, Kentoffio K, Niyonzima JB (2015). Remoteness and maternal and child health service utilization in rural Liberia: a population-based survey. J Glob Health.

[8] Shah JA, Emina JBO, Eckert E, Ye Y (2015). Prompt access to effective malaria treatment among children under five in sub-Saharan Africa: a multi-country analysis of national household survey data. Malar J.

[9] Rebuilding Basic Health Services. Behavior change communication RBHS End-of-Project Report, October 2010–October 2014. Monrovia: Rebuilding Basic Health Services; 2014.

[10] Simba DO, Warsame M, Kakoko D, Mrano Z, Tomson G, Premji Z (2010). Who get prompt access to artemisinin-based combination therapy? A prospective community-based study in children from rural Kilosa, Tanzania. PLoS ONE.

[11] Killian A, Lawford H, Ujuju CN, Abeku TA, Nwokoio E, Okoh F (2016). The impact of behavior change communication on the use of insecticide treated nets: a secondary analysis of ten post-campaign surveys from Nigeria. Malar J.

[12] Ricotta EE, Boulay M, Ainslie R, Babalola S, Fotheringham M, Koenker H (2015). The use of mediation analysis to assess the effects of a behavior change communication strategy on bed net ideation and household universal coverage in Tanzania. Malar J.

[13] Liu JX, Sepideh M (2016). Evaluation of SMS reminder messages for altering treatment adherence and health seeking perceptions among malaria care-seekers in Nigeria. Health Policy Plan.

[14] Fatungase KO, Amoran OE, Alausa KO (2012). The effect of health education intervention on the home management of malaria among the caregivers of children aged under 5 years in Ogun State, Nigeria. Eur J Med Res.

[15] Dillip S, Alba A, Hetzel M, Mayumana I, Mshana C, Makemba A (2010). Improvements in access to malaria treatment in Tanzania following community, retail sector and health facility interventions—a user perspective. Malar J.

[16] Kazembe L, Appleton C, Kleinschmidt I (2007). Choice of treatment for fever at household level in Malawi: examining spatial patterns. Malar J.

[17] Ewing VL, Rolhurst R, Kapinda A, Richards E, Terlouw DJ, Lalloo DG (2016). Increasing understanding of the relationship between geographic access and gendered decision-making power for treatment-seeking for febrile children in the Chikwawa district of Malawi. Malar J.

[18] Getahun A, Deribe K, Deribew A (2010). Determinants of delay in malaria treatment-seeking behavior for under-five children in south-west Ethiopia: a case control study. Malar J.

[19] Kassile T, Lokina R, Mujinja P, Mmbando B (2014). Determinants of delay in care seeking among children under five with fever in Dodoma region, central Tanzania: a cross-sectional study. Malar J.

[20] Chukwuocha UM, Okpanma AC, Nwakwuo GC, Dozie INS (2014). Determinants of delay in seeking malaria treatment for children inder-five years in parts of south eastern Nigeria. J Community Health.

[21] Mitiku I, Assefa A (2017). Caregivers’ perception of malaria and treatment-seeking behavior for under five children in Mandura District, West Ethiopia: a cross-sectional study. Malar J.

[22] Nyenswah TG, Kateh F, Bawo L, Massaquoi M, Gbanyan M, Fallah M (2016). Ebola and its control in Liberia, 2014–2015. J Emerg Infect Dis.

[23] Furuse Y, Fallah M, Oshitani H, Kituyi L, Mahmoud N, Musa E (2016). Analysis of patient data from laboratories during the Ebola virus disease outbreak in Liberia, April 2014 to March 2015. PLoS Negl Trop Dis.

